# Sequencing, Annotation and Analysis of the Syrian Hamster (*Mesocricetus auratus*) Transcriptome

**DOI:** 10.1371/journal.pone.0112617

**Published:** 2014-11-14

**Authors:** Nicolas Tchitchek, David Safronetz, Angela L. Rasmussen, Craig Martens, Kimmo Virtaneva, Stephen F. Porcella, Heinz Feldmann, Hideki Ebihara, Michael G. Katze

**Affiliations:** 1 Department of Microbiology, University of Washington, Seattle, Washington, United States of America; 2 Laboratory of Virology, Division of Intramural Research, National Institute of Allergy and Infectious Diseases, National Institutes of Health, Rocky Mountain Laboratories, Hamilton, Montana, United States of America; 3 Genomics Unit, Research Technologies Section, National Institute of Allergy and Infectious Diseases, National Institutes of Health, Rocky Mountain Laboratories, Hamilton, Montana, United States of America; 4 Washington National Primate Research Center, University of Washington, Seattle, Washington, United States of America; Ecole Normale Supérieure de Lyon, France

## Abstract

**Background:**

The Syrian hamster (golden hamster, *Mesocricetus auratus*) is gaining importance as a new experimental animal model for multiple pathogens, including emerging zoonotic diseases such as Ebola. Nevertheless there are currently no publicly available transcriptome reference sequences or genome for this species.

**Results:**

A cDNA library derived from mRNA and snRNA isolated and pooled from the brains, lungs, spleens, kidneys, livers, and hearts of three adult female Syrian hamsters was sequenced. Sequence reads were assembled into 62,482 contigs and 111,796 reads remained unassembled (singletons). This combined contig/singleton dataset, designated as the Syrian hamster transcriptome, represents a total of 60,117,204 nucleotides. Our *Mesocricetus auratus* Syrian hamster transcriptome mapped to 11,648 mouse transcripts representing 9,562 distinct genes, and mapped to a similar number of transcripts and genes in the rat. We identified 214 quasi-complete transcripts based on mouse annotations. Canonical pathways involved in a broad spectrum of fundamental biological processes were significantly represented in the library. The Syrian hamster transcriptome was aligned to the current release of the Chinese hamster ovary (CHO) cell transcriptome and genome to improve the genomic annotation of this species. Finally, our Syrian hamster transcriptome was aligned against 14 other rodents, primate and laurasiatheria species to gain insights about the genetic relatedness and placement of this species.

**Conclusions:**

This Syrian hamster transcriptome dataset significantly improves our knowledge of the Syrian hamster's transcriptome, especially towards its future use in infectious disease research. Moreover, this library is an important resource for the wider scientific community to help improve genome annotation of the Syrian hamster and other closely related species. Furthermore, these data provide the basis for development of expression microarrays that can be used in functional genomics studies.

## Introduction

The Syrian hamster (golden hamster, *Mesocricetus auratus*) has recently been used as an experimental rodent model for important infectious diseases including Ebola and other viral hemorrhagic fevers [1]–[8]. For instance, Syrian hamsters infected with mouse-adapted Ebola virus (EBOV) manifest many of the clinical and pathological findings observed in EBOV-infected non-human primates (NHPs) and humans, including systemic viral replication, suppression of the innate immune response, an uncontrolled inflammatory response, and disseminated intravascular coagulation syndrome [9]. The Syrian hamster is emerging as a promising model for leishmaniasis [10] and dyslipidaemia research [11], [12]. The Syrian hamster is also an important animal model in neurosciences research [13], [14]. For instance, this species has been widely used in the studies of circadian rhythms [15], cardiomyopathy [16], aggression [17], reproduction [18], and sensory systems [19].

Genotyping of *Mesocricetus auratus* is currently under way at the Broad Institute (NCBI-BioProject accession: PRJNA77669) but not yet published. So far, only 860 cDNA sequences from the Syrian hamster are available in the NCBI-dbEST database [20], where 728 sequences have been collected in the context of testis organs [21] and 125 sequences have been collected in the context of embryonic cells [22]. More recently, while Schmucki et al. analyzed the liver transcriptome of the Syrian hamster with a focus on lipid metabolism [23] the data is not publicly available as of this writing.

Drafts of the genome and transcriptome of Chinese hamster ovary (CHO) cells have recently been published [24], [25], although it should be noted that CHO cells represent cells in an immortalized condition and therefore will likely contain genetic mutations not present in natural conditions. The current release of the CHO cell draft genome is composed of 109,152 scaffolds and 265,786 contigs representing a total length of 2,318,115,958 nucleotides. Preliminary gene annotation of the CHO cell genome was performed using vertebrate experimental data and cross-species comparisons. The current release of the CHO cell transcriptome comprises 121,636 transcript fragments representing a total length of 179,731,611 nucleotides. More recently, Lewis et al. compared the genome of CHO cells and the genome of the Chinese hamster obtained from tissues, and they showed a significant proximity between these different conditions [26]. Further efforts will be continued regarding the update of the CHO and Chinese hamster genomes and transcriptomes.

The aims of our study were: (i) to provide to the scientific community a large panel of annotated mRNA sequences from the *Mesocricetus auratus* transcriptome; (ii) to provide new biological insights and knowledge about the *Mesocricetus auratus* species; and (iii) to use this data to allow the design of a future gene expression microarray. Here we sequenced a normalized 3′ mRNA fragment primed cDNA library produced from pooled RNA isolated from the major organs of adult female Syrian hamsters following strategies in common-use described elsewhere [27], [28]. We reasoned that pooling a large variety of different organs of animals will provide a large pool of mRNA fragments to sequence and annotate. Sequencing reads were de novo assembled into contigs. The combined contig and unassembled read (singleton) dataset, designated as the Syrian hamster transcriptome, was annotated based on the mouse and rat transcriptomes. We identified the most highly covered and the most highly expressed transcripts in our Syrian hamster transcriptome and performed a functional enrichment analysis to identify which canonical pathways and biological functions were most significantly represented. In order to contribute to the annotation efforts of the Chinese hamster species, we aligned our Syrian hamster transcriptome to the current version of the CHO cell genome and transcriptome. Finally, we aligned our Syrian hamster transcriptome to 14 other primate species and analyzed the genomic divergence of our transcripts in order to gain insights into the genomic evolution of the Syrian hamster.

## Results

### Sample collection and sequencing of a cDNA library produced from female Syrian hamster organs

**Figure 1:**
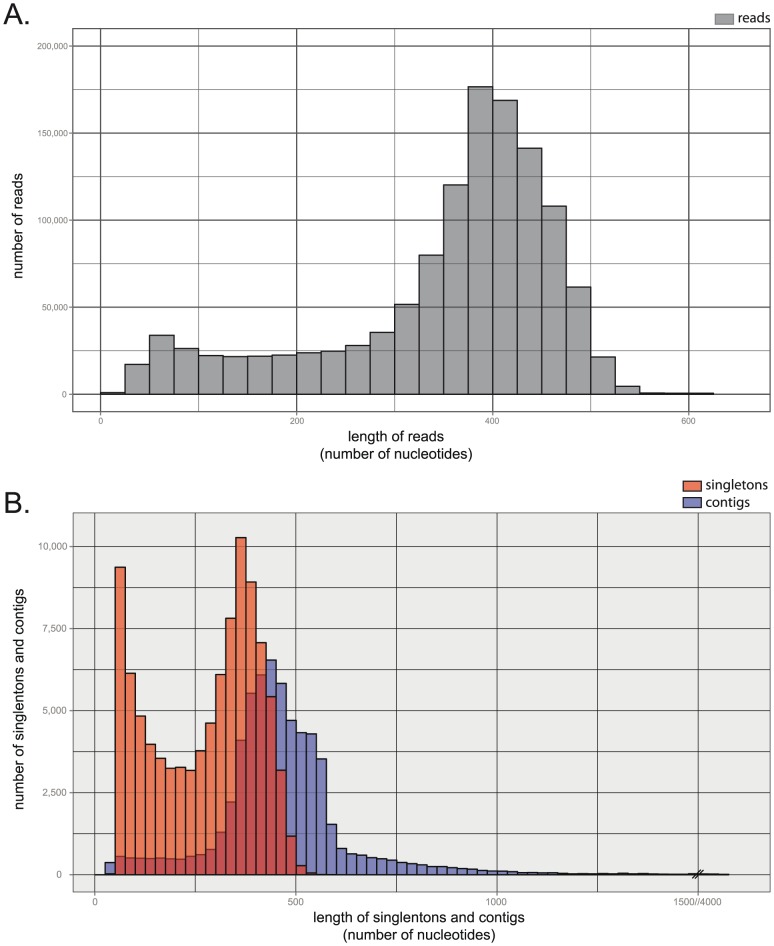
Histograms showing the length distribution of the reads and the length distribution of the singletons and contigs. (A) The length distribution of the reads is shown in a gray histogram. Bins of the histogram have been set to 50 nucleotides. The lengths of the reads range from 40 to 631, with a median length of 387 and a mean length of 352. The reads represents a total of 426,683,712 nucleotides bases. (B) The length distribution of the 111,796 singletons is shown in a red histogram while the length distribution of the 62,482 contigs is shown in a blue histogram. Bins of the histograms have been set to 25 nucleotides. The lengths of the singleton sequences range from 50 to 614, with a median length of 187 and a mean length of 265. The lengths of the contig sequences range from 50 to 4,054, with a median length of 473 and a mean length of 487. Our Syrian hamster transcriptome represents a total of 60,117,204 nucleotides bases.

The brains, lungs, spleens, kidneys, livers, and hearts were collected from three adult female Syrian hamsters. Total RNAs were isolated, pooled, and contaminating genomic DNA removed. Following adaptor ligation, cDNAs were 3′ fragment-sequenced on a Roche 454 GS FLX Titanium instrument. The sequencing generated 1,283,840 reads with an average length of 344 bases. Reads were trimmed for quality and reads shorter than 40 bases were discarded, resulting in 1,212,395 sequence reads available for further assembly and analysis. **Figure 1A** shows the length distribution of reads before assembly. Consistent with most of the publicly available transcriptome libraries [29], we observed that our reads ranged between 200 and 600 nucleotides in length.

### Library assembly

Quality-filtered reads were assembled into contigs. Resulting contigs and unassembled reads (singletons) were quality filtered and contigs or singletons shorter than 50 bases were discarded. Among the 1,212,395 reads, 62,482 contigs and 111,796 singletons were generated. **Figure 1B** shows the length distributions of the 174,278 combined contig/singleton dataset. The lengths of the singletons ranged from 50 to 614, with a median length of 187.50 bases. The lengths of the contigs ranged from 50 to 4,054, with a median length of 473.50 bases. We observed that most of the reads ranging between 75 and 400 nucleotides were assembled. Short reads are subject to noise and have low quality scores, making them more difficult to assemble. On the other hand, larger reads are difficult to assemble in this context because our library was targeted against 3′ mRNA priming. The final dataset (contigs plus singletons) represents a total of 60,117,204 nucleotides and is designated as the Syrian hamster transcriptome.

### Library annotation

The Syrian hamster transcriptome was aligned to the mouse and rat transcriptome references (**Table 1**). Amongst the 174,278 contigs and singletons, 41,651 (23.90%) were significantly aligned (expected value cutoff of 10) to the mouse transcriptome and 26,258 (15.07%) were significantly aligned to the rat transcriptome. Of these, 11,648 transcripts (representing 9,562 genes) contained functional annotation in the mouse transcriptome, and 7,223 transcripts (representing 7,137 genes) were functionally annotated in the rat transcriptome (**Table 1**). Therefore, 11,648 Syrian sequence fragments or transcripts are now annotated by way of homology with the mouse genome.

**Table 1:** Transcriptome references and alignment statistics.

Species name	# of genes	# of transcripts	# and % of alignments	# and % of mapped genes	# and % of mapped transcripts
Mouse (Mus musculus)	38,293	92,484	41,651 (23.90%)	9,562 (22.96%)	11,648 (12.59%)
Rat (Rattus norvegicus)	26,405	29,189	26,258 (15.07%)	7,137 (27.18%)	7,223 (24.75%)
Chinese Hamster Ovary cells (Cricetulus griseus)	NA	121,636*	7,845 (4.50%)	NA	4,390 (3.61%)
Chimpanzee (Pan troglodytes)	28,012	29,160	2,884 (1.65%)	1,631 (56.55%)	1,643 (5.63%)
Ferret (Mustela putorius furo)	23,811	23,963	16,169 (9.28%)	4,169 (25.78%)	4,187 (17.47%)
Gorilla (Gorilla gorilla gorilla)	29,216	35,727	8,319 (4.77%)	2,733 (32.85%)	2,735 (7.66%)
Guinea pig (Cavia porcellus)	25,028	26,129	15,014 (8.61%)	4,050 (26.97%)	4,155 (15.90%)
Human (Homo sapiens)	62,316	213,551	23,020 (13.21%)	5,409 (23.50%)	7,254 (3.40%)
Kangaroo rat (Dipodomys ordii)	26,405	29,189	2,103 (1.21%)	1,252 (59.53%)	1,252 (4.29%)
Macaque (Macaca mulatta)	30,246	44,725	13,792 (7.91%)	3,804 (27.58%)	4,163 (9.31%)
Orangutan (Pongo abelii)	28,443	29,447	15,331 (8.80%)	3,929 (25.63%)	3,952 (13.42%)
Pig (Sus scrofa)	25,322	30,586	10,910 (6.26%)	2,978 (27.30%)	3,051 (9.98%)
Pika (Ochotona princeps)	23,028	23,028	1,575 (0.90%)	989 (62.79%)	989 (4.29%)
Rabbit (Oryctolagus cuniculus)	23,394	28,188	4,344 (2.49%)	1,946 (44.80%)	2,007 (7.12%)
Shrew (Sorex araneus)	19,134	19,139	1,330 (0.76%)	759 (57.07%)	759 (3.97%)
Squirrel (Ictidomys tridecemlineatus)	22,398	23,572	7,730 (4.44%)	2,723 (35.23%)	2,733 (11.59%)
Tree Shrew (Tupaia belangeri)	20,820	20,824	1,786 (1.02%)	1,091 (61.09%)	1,091 (5.24%)

For each transcriptome reference used in this study, the name of the species, the number of genes available, and the number of transcripts available are indicated.

*The number of available transcripts indicated for the Chinese hamster ovary cells represents the number of available transcript fragments available and not the number of distinct transcripts. Moreover, for each transcriptome reference used in this study, the number of aligned contigs and singletons, the number of mapped transcripts and the number of mapped genes are indicated. The percentages of mapped transcripts and mapped genes relative to the total number of transcripts and genes available on the transcriptome references are provided. Moreover the percentage of alignments relative to the total number of contigs and singletons in our library (174,278) is also provided.

**Figure 2:**
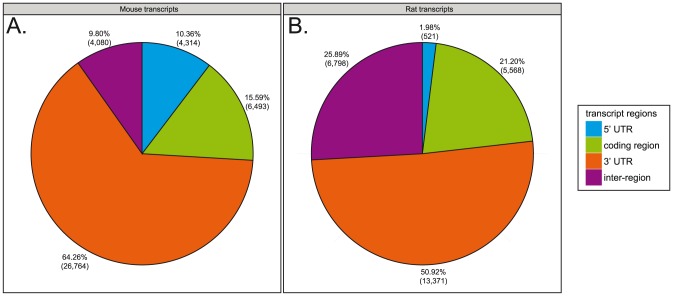
Pie diagrams showing the alignment positions of the contigs and singletons on the mouse and rat transcript regions. (A) Pie diagram showing the distribution of alignment positions of the 41,651 contigs and singletons on the mouse transcripts regions (5′ UTR, coding region, 3′ UTR, or inter-region). (B) Pie diagram showing the distribution of alignment positions of the 26,258 contigs and singletons on the rat transcripts regions. For each species and transcript region the number and percentage of aligned sequences are indicated.

We also investigated the positioning of the mRNA encoded contigs and singletons of our Syrian hamster transcriptome against other species' different transcript regions such as, 5′ untranslated regions (5′ UTR), coding regions, or 3′ untranslated regions (3′ UTR). With respect to the mouse transcriptome reference, 4,314 fragments of our Syrian hamster transcriptome (10.36%) aligned to 5′ UTRs, while 6,493 fragments of our dataset (15.59%) aligned to coding regions. In addition, 26,764 fragments of the Syrian hamster transcriptome (64.26%) aligned to 3′ UTRs (**Figure 2A**). A further 4,080 fragments of the Syrian hamster transcriptome (9.80%) aligned between 5′ UTRs, coding regions, and 3′ UTRs of the mouse transcriptome. Based on the rat transcriptome reference, 521 fragments of the Syrian hamster transcriptome (1.98%) aligned to 5′ UTRs while, 5,568 fragments of the Syrian hamster transcriptome (21.20%) aligned to coding regions. In addition, 13,371 fragments of the Syrian hamster transcriptome (50.92%) aligned to 3′ UTRs (**Figure 2B**). Finally, a total of 6,798 fragments of the Syrian hamster transcriptome (25.89%) aligned between 5′ UTRs, coding regions, and 3′ UTRs of the rat transcriptome. As expected from the experimental design of our library, the majority of our Syrian hamster transcriptome sequences aligned to 3′ UTRs of mouse and rat annotated transcripts.

Regarding the publicly available mouse and rat genome datasets, 45,804 of our Syrian hamster transcriptome fragments aligned to either the mouse or rat transcriptomes, and 22,105 of these same sequences aligned to both transcriptomes simultaneously, suggesting commonly occurring transcripts. Our Syrian hamster transcriptome dataset was 65.10% similar to the mouse transcriptome and 64.46% similar to the rat transcriptome. These similarities increased to 74.48% and 74.26% for the mouse and rat respectively, when comparisons were restricted to coding regions-only within those two reference genomes.

In the mouse genome, we found that 214 of those transcripts mapped at 90% of their lengths to either contigs or singletons in the Syrian hamster transcriptome (**Table S1**). Among these highly covered mouse transcripts were genes associated with a range of cellular activities involving, but not limited to inflammation, cell death, metabolism, and initiation of translation. These results suggest that highly covered transcripts, representative of a wide variety of cellular processes, were obtained through our methodology.

### Over-expressed sequence reads and over-represented canonical pathways

In order to obtain further biological insight into our Syrian hamster transcriptome, we next identified over-expressed genes based on the number of individual reads that mapped to mouse-annotated genes (**Table 2**). We found that 20 mouse genes contained at least 600 x read depth, and 49 mouse genes contained at least 500 fold read depth.

**Table 2:** List of the top 50 expressed genes in the library.

Ensembl Gene ID	Associated Gene Name	Description	Count
ENSMUSG00000028647	Mycbp	c-myc binding protein	1120
ENSMUSG00000020594	Pum2	pumilio 2 (Drosophila)	1017
ENSMUSG00000008575	Nfib	nuclear factor I/B	945
ENSMUSG00000022010	Tsc22d1	TSC22 domain family, member 1	895
ENSMUSG00000062078	Qk	quaking	861
ENSMUSG00000078578	Ube2d3	ubiquitin-conjugating enzyme E2D 3	795
ENSMUSG00000026621	Mosc1	MOCO sulphurase C-terminal domain containing 1	710
ENSMUSG00000028161	Ppp3ca	protein phosphatase 3, catalytic subunit, alpha isoform	707
ENSMUSG00000028790	Khdrbs1	KH domain containing, RNA binding, signal transduction associated 1	695
ENSMUSG00000006740	Kif5b	kinesin family member 5B	684
ENSMUSG00000031627	Irf2	interferon regulatory factor 2	682
ENSMUSG00000036781	Rps27l	ribosomal protein S27-like	660
ENSMUSG00000026655	Fam107b	family with sequence similarity 107, member B	658
ENSMUSG00000006373	Pgrmc1	progesterone receptor membrane component 1	652
ENSMUSG00000060961	Slc4a4	solute carrier family 4 (anion exchanger), member 4	641
ENSMUSG00000024750	Zfand5	zinc finger, AN1-type domain 5	639
ENSMUSG00000028788	Ptp4a2	protein tyrosine phosphatase 4a2	634
ENSMUSG00000019943	Atp2b1	ATPase, Ca++ transporting, plasma membrane 1	605
ENSMUSG00000097347	AC121292.1		603
ENSMUSG00000004980	Hnrnpa2b1	heterogeneous nuclear ribonucleoprotein A2/B1	600
ENSMUSG00000093904	Tomm20	translocase of outer mitochondrial membrane 20 homolog (yeast)	593
ENSMUSG00000068823	Csde1	cold shock domain containing E1, RNA binding	586
ENSMUSG00000020315	Spnb2	spectrin beta 2	579
ENSMUSG00000068798	Rap1a	RAS-related protein-1a	579
ENSMUSG00000020390	Ube2b	ubiquitin-conjugating enzyme E2B	570
ENSMUSG00000026064	Ptp4a1	protein tyrosine phosphatase 4a1	570
ENSMUSG00000020053	Igf1	insulin-like growth factor 1	569
ENSMUSG00000027706	Sec62	SEC62 homolog (S. cerevisiae)	553
ENSMUSG00000064373	Sepp1	selenoprotein P, plasma, 1	549
ENSMUSG00000014956	Ppp1cb	protein phosphatase 1, catalytic subunit, beta isoform	538
ENSMUSG00000007850	Hnrnph1	heterogeneous nuclear ribonucleoprotein H1	536
ENSMUSG00000031207	Msn	moesin	518
ENSMUSG00000020152	Actr2	ARP2 actin-related protein 2	515
ENSMUSG00000022261	Sdc2	syndecan 2	514
ENSMUSG00000047187	Rab2a	RAB2A, member RAS oncogene family	512
ENSMUSG00000004936	Map2k1	mitogen-activated protein kinase kinase 1	510
ENSMUSG00000026576	Atp1b1	ATPase, Na+/K+ transporting, beta 1 polypeptide	506
ENSMUSG00000022234	Cct5	chaperonin containing Tcp1, subunit 5 (epsilon)	504
ENSMUSG00000001175	Calm1	calmodulin 1	502
ENSMUSG00000069662	Marcks	myristoylated alanine rich protein kinase C substrate	490
ENSMUSG00000017776	Crk	v-crk sarcoma virus CT10 oncogene homolog (avian)	484
ENSMUSG00000038014	Fam120a	family with sequence similarity 120A	484
ENSMUSG00000036478	Btg1	B cell translocation gene 1, anti-proliferative	483
ENSMUSG00000027177	Hipk3	homeodomain interacting protein kinase 3	478
ENSMUSG00000043991	Pura	purine rich element binding protein A	474
ENSMUSG00000022283	Pabpc1	poly(A) binding protein, cytoplasmic 1	471
ENSMUSG00000031342	Gpm6b	glycoprotein m6b	471
ENSMUSG00000050608	Minos1	mitochondrial inner membrane organizing system 1	471
ENSMUSG00000018446	C1qbp	complement component 1, q subcomponent binding protein	469
ENSMUSG00000026568	Mpc2	mitochondrial pyruvate carrier 2	461

For each of the top 50 expressed genes in the library, based on the mouse annotations, the Ensembl mouse gene identified, the associated gene name, description, and the number of count (number of time that the genes have been mapped by the reads) are indicated.

Most of the mouse genes showing high read depth were annotated as being involved in fundamental cellular processes such as cell morphology and organization, cell cycle progression, cell function and maintenance, transcription, protein synthesis and turnover, cell death, and molecular transport. Genes associated with cell type or tissue-specific functions were not significantly over-represented, consistent with our method of generating cDNA reads from pooled, multiple organ tissues. Our aim in this study was to sequence and annotate a large number of hamster mRNA 3′ fragments as a preliminary effort towards generation of an expression array, our observation that the distribution of reads were across common cellular functions, suggests our assembly is not overly biased against a specific cell or tissue type.

**Figure 3:**
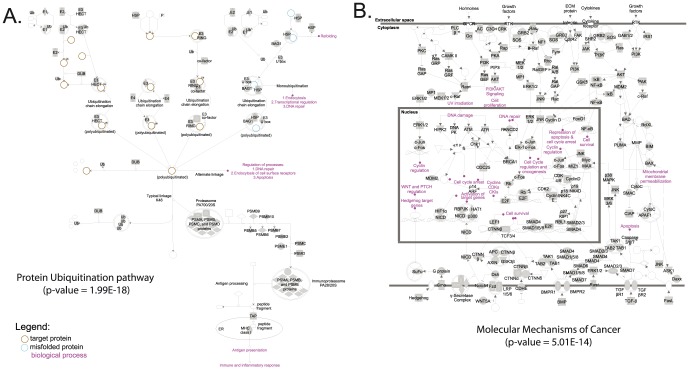
Schematic representation of the top two over-represented canonical pathways in our transcriptome assembly. (A) Representation of the “Protein Ubiquitination” canonical pathway. (B) Representation of the “Molecular Mechanisms of Cancer” canonical pathway. Both pathways have been generated based on mouse annotations. Transcripts involved in these pathways are indicated by different node shapes and associations are indicated by different edge shapes. Legends for the different nodes and edges are given in **Figure S1**. For both pathways, transcripts present in our library are indicated in gray. Associated p-values showing the statistical over-representation significance of the canonical pathways are also indicated.

We also performed a functional enrichment analysis of our Syrian hamster transcriptome. Based on the list of 9,562 mouse genes that were mapped to our contigs and singletons, we identified the over-represented canonical pathways in our library (**Table 3**). “Protein ubiquitination” (**Figure 3A**, p-value  = 1.99E-18) and “molecular mechanisms of cancer” (**Figure 3B**, p-value  = 5.01E-14) were the two most over-represented canonical pathways. However, there was also significant enrichment of many other canonical pathways related to biochemical, cellular, and disease-associated cellular processes. These included a multitude of signaling pathways, including RhoGTPase, protein kinase A, integrin, Rac, ERK/MAPK, mTOR, PI3K/Akt, PTEN, insulin, WNT/b-catenin, growth factor (VEGF, NGF, HGF, FGF, GM-CSF), and cellular junction signaling pathways. All of these pathways are biologically essential for intra- and intercellular communication and have known pleiotropic effects on transcription and translation, cellular proliferation, development, differentiation, cytoskeletal dynamics, cellular morphology, cell death, metabolism, and host responses to stress or infection. Consistent with this data, we also observed enrichment of functional categories associated with these biological activities (**Table 3**). The biological functions associated with “cardiovascular system development and function” (p-values range from 1.05E-03 to 4.15E-17) and “nervous system development and function” (p-values range from 1.29E-03 to 1.46E-19) were statistically over-represented.

**Table 3:** Functional enrichment of the mouse genes mapped by our transcriptome assembly.

Rank	Biological Function [p-value range]	Canonical pathway (p-value)
1	Organismal Surviva [1.11E-03 – 4.03E-26]	Protein Ubiquitination Pathway (1.99E-18)
2	Nervous System Development and Function [1.29E-03 – 1.46E-19]	Molecular Mechanisms of Cancer (5.01E-14)
3	Organ Morpholog [1.32E-03 – 4.20E-19]	Integrin Signaling (3.16E-13)
4	Tissue Morphology [1.08E-03 – 1.07E-18]	EIF2 Signaling (3.98E-12)
5	Cardiovascular System Development and Function [1.05E-03 – 4.15E-17]	Epithelial Adherens Junction Signaling (2.51E-11)

List of the top 5 biological functions and the top 5 canonical pathways found as statistically over-represented based on the list of 9,546 mouse genes mapped by our transcriptome assembly. The range of p-values is indicated for the biological functions and the p-value is indicated for each canonical pathways.

### Comparison with the Chinese hamster species

In order to contribute to the annotation efforts for the Chinese hamster (*Cricetulus griseus*) species, we aligned our Syrian hamster transcriptome to the current draft versions of the CHO cell genome and its transcriptome (**Table 1**).

We found that 7,845 fragments in our Syrian hamster transcriptome aligned to the CHO cell transcriptome (**Table 1**) and 85,652 aligned to the CHO cell genome (**Table S2**). On the other hand, 4,390 transcript fragments from the CHO cell dataset mapped to the Syrian hamster transcriptome (**Table 1**). Our aligned Syrian hamster transcriptome showed 85.14% similarity with the CHO cell transcriptome, an expectedly higher value than what we saw for the same comparison with the mouse and rat transcriptomes.

### Cross-species comparison

In order to obtain further insights about the genomic evolution of the Syrian hamster we aligned our Syrian hamster transcriptome to 14 other transcriptomes, all of which are publicly available on the Ensembl database [30] (**Table 1**). This compendium of transcriptome references included the human (*Homo sapiens*), chimpanzee (*Pan Troglodytes*), gorilla (*Gorilla gorilla gorilla*), macaque (*Macaca mulatta*), and orangutan (*Pongo abelii*) sequences, as well as the ferret (*Mustela putorius furo*), guinea pig sequences (*Cavia porcellus*), and pig (*Sus scrofa*). As expected, the greatest number of aligned sequences occurred with the mouse and rat species transcriptomes (**Table 1**). The human and the non-human primate species also showed high numbers of aligned sequences, possibly due to the current high quality assembly and annotation of those genomes. The CHO, ferret, pig, rabbit (*Oryctolagues cuniculus*), and squirrel (*Ictidomys tridecemlineatus*) species showed intermediary numbers of aligned sequences, while the guinea pig, kangaroo rat (*Dipodomys ordii*), pika (*Ochotona princeps*), shrew (*Sorex araneus*) and tree shrew (*Tupaia belangeri*) had the lowest numbers of aligned sequences. Of 174,278 Syrian hamster transcriptome fragments 50,433 aligned to at least one transcript reference while 61 fragments from our dataset aligned in common across all of these transcriptome references. Importantly, 76,175 of our Syrian hamster transcriptome fragments did not align to any of the 17 transcriptomes tested, nor to the CHO cell genome. It is important to note that some of the variability seen in our transcriptome comparisons may be due to differences in genome quality, assembly and annotation for the reference genomes tested.

**Figure 4:**
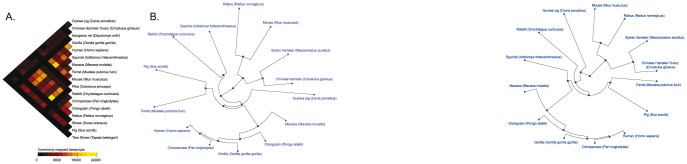
Distogram showing the commonly mapped transcripts and phylogenetic tree showing the divergences amongst the different species. (A) Distogram showing the number of transcripts commonly mapped by the Syrian hamster transcriptome between the different species used in this study. Each cell of the distogram represents the number of transcripts commonly mapped by two different species using a gradient color. (B) Phylogenetic tree showing the genomic divergence between a subset of the different species used in this study. Each leaf of the tree represents a different species and the distances of the edges are proportional to the genomic distances between the species. Genomic distances have been calculated based on the list of 611 Syrian hamster contigs and singletons that have been commonly aligned on the transcriptome references of the 13 species having the highest number of commonly aligned sequences.

**Figure 4A** is a distogram showing the results of our analysis of transcript sequences shared in common. The kangaroo rat, pika, shrew, and tree shrew had the lowest amount of commonly aligned sequences, amongst themselves and with the other species. The mouse and rat species showed the highest number of aligned sequences, presumably because of both their relatedness and genome quality/completeness.

We then investigated the evolutionary divergence between the Syrian hamster and the 13 species with the largest numbers of mapped sequences and the largest degrees of shared sequences (i.e. excluding the pika, kangaroo rat, shrew, tree shrew). We found that 611 transcriptomic fragments (**Table S3**) have been significantly aligned on the transcriptome references of these 13 most related species and we constructed a phylogenetic tree (**Figure 4B**).The Syrian hamster transcriptome branched most closely with the CHO genome as expected. The mouse and rat transcriptome clustered together and close to the Syrian hamster and CHO cluster, as expected. All the primate species formed a super group, while the ferret and pig transcriptomes clustered together as the rabbit and squirrel transcriptomes. Consistent with a recently published study [31], we observed that the genomic divergence between the Syrian and Chinese hamsters is comparable to the divergence seen between the rat and mouse. Also, as expected, we observed that the Guinea pig does not cluster with the rodent species [32], [33].

## Discussion

Here we present the assembly and analysis of a Syrian hamster transcriptome derived from the pooled RNAs from brains, lungs, spleens, kidneys, livers, and hearts of three adult females. The 3′ poly-T primed cDNAs that were sequenced on a long read-format Roche 454 were assembled into contigs, such that 22,105 of these contigs or singletons were annotated based on homology with both the mouse and rat transcriptomes, while 45,804 contigs or singletons were annotated based upon homology to one or the other mouse or rat transcriptomes. We identified 214 quasi-complete transcript sequences based on homology with mouse mRNAs and their annotations. In addition, we aligned our Syrian hamster transcriptome to the CHO cell transcriptome in order to further annotate our hamster species, and we observed a transcriptome similarity of 85.14% between the two.

When compared to a large compendium of transcriptome references, comprised of rodent, primate, and laurasiatheria species, using 661 Syrian hamster transcriptome fragments that aligned in common, the Syrian hamster transcriptome was found to be evolutionarily closest to the CHO genome and in close proximity to the mouse and rat species. The branch pattern and branch length between the Syrian and Chinese hamster transcriptomes was found to be similar to that observed between the mouse and the rat species. This observation was also described by Ryu et al. [31], but those previous efforts focused on mitochondrial gene sequences for their phylogeny analysis.

In the Syrian hamster transcriptome, we were able to identify a number of genes involved in a broad spectrum of fundamental biological processes. In addition to the 214 quasi-complete transcripts, identified based on mouse annotations and the most highly expressed transcripts, functional analysis of the entire set of sequence fragments in the Syrian hamster transcriptome that mapped to mouse genes revealed that a number of critical biological pathways are well-represented, including many related to key processes that are potentially perturbed or induced during infection. Among the most significantly enriched canonical pathways were several involved with protein synthesis, turnover, and antigen processing (protein ubiquitination, EIF2 signaling), metabolism and stress responses (mitochondrial dysfunction, NRF2-mediated oxidative stress response, PI3K/Akt, and mTOR signaling), and inflammatory and immune responses (production of NO and reactive oxygen species by macrophages, CXCR4 signaling, IL-1 signaling, and IL-3 signaling). The aim of this study was to collect and annotate a large panel of transcripts regardless of tissue origin. These observations suggest that we have generated a representative transcriptome of the Syrian hamster. Therefore this transcriptome data could be used to generate a biologically meaningful first-generation expression DNA microarray for analysis of Syrian hamster response to disease, including those infectious agents known to alter immune and pro-inflammatory responses. Mechanisms of transcriptome regulation in the Syrian hamster, by way of these important pathways can now be monitored and analyzed further.

Only ∼20% of the fragments in the Syrian hamster transcriptome aligned to the mouse and rat transcriptomes and even less aligned to the CHO cell transcriptome. This low percentage is due in part to species specificity, alignment stringency, but also to the fact that transcriptome references are far from being completely known and annotated. For instance, some classes of non-coding transcripts are now increasingly recognized as major components of regulation, and are widely expressed, but are poorly characterized and annotated. The transcriptome references that we used mainly contain known and annotated transcripts and our assembly may contain many expression contigs and singletons currently unknown and un-annotated in these other genomes.

The CHO cell genome is a useful tool for further improving the quality of our Syrian hamster transcriptome annotation for functional genomics work [24], [25]. CHO cells have been used in a variety of genetic, cell biology, and pharmacology studies. They also are the mammalian cell line of choice for producing large quantities of recombinant proteins in large amounts or in or industrial laboratory settings. Although Chinese and Syrian hamsters are phylogenetically distinct within the rodent subfamily *Cricetinae* [34], [35], our data confirm that they are more closely related to one another as compared to other muroid rodents.

Through our work, we have increased the number of contig sequences available in the public domain for the Syrian hamster from 860 to 174,278, where 50,433 (28.93% of the Syrian hamster transcriptome) aligns to at least one transcriptome reference. Moreover, 85,652 (49.14% of the Syrian hamster transcriptome) fragments have aligned to the draft CHO genome, leading to an overall total of 98,103 (56.29%) annotated Syrian hamster transcripts. As a note, the work performed by Schmucki et al in [23] focused on transcriptome analysis of lipid metabolism in the golden hamster liver, and no contigs or other sequences have been released to the public domain to date.

With additional funding, future plans are in place for Illumina-based RNA sequencing using paired-end technology to add and improve on our current contig assembly. These efforts will improve our coverage of the Syrian hamster transcriptome, as well as permit more comprehensive and robust phylogenetic comparisons with other species. These combined efforts will lead to a better understand of the Syrian hamster transcriptome under a variety of infectious agent models related to human disease and pathogenesis.

## Conclusions

The Syrian hamster is becoming an increasingly popular model for a variety of diseases, in particular, diseases known to infect non-human primates and humans. This Syrian hamster transcriptome discussed here represents a critical step forward in providing the tools necessary for advancing functional genomics in this important animal model.

## Material and Methods

### Animal housing

All hamsters were housed in individually ventilated cages (IVCs). All hamsters are co-housed, unless scientifically justified and approved by the Institutional Animal Care and Use Committee (IACUC) or deemed necessary for veterinary reasons. Housing density is determined by the guidelines outlined in the Guide for the Care and Use of Laboratory Animals and the Association for the Assessment and Accreditation of the Laboratory Animal Care, International (AAALAC). Food and sterile or acidified water were provided ad libitum. Hamster diets were consist of pellets containing a variety of foods such as grains and dried vegetables along with some seeds. Water was provided by either water bottles or water pouches. The light/dark cycle was 14 hours light, 10 hours dark.

### RNA extraction

Three adult female Syrian hamsters were euthanized (exsanguinated while under isoflurane sedation) and six tissues – liver, lung, heart, brain, kidney, and spleen – were harvested from each hamster. All animal studies conformed to the guidelines set forth by the National Institutes of Health (NIH) and were reviewed and approved by the Institutional Animal Care and Use Committee (IACUC) at Rocky Mountain Laboratories, Division of Intramural Research, National Institute of Allergy and Infectious Diseases, NIH. One hundred mg of hamster tissue was homogenized with a Qiagen TissueLyzer II (Qiagen, Valencia, CA) in 1 mL Trizol (Invitrogen, Carlsbad, CA) following manufacturer's recommendations. To each aliquot 200 µL of 1-bromo-3-chloropropane (Sigma-Alrich) was added, the mixture was vortexed for 15 seconds and centrifuged at 4°C at 16,000x for 15 minutes. The aqueous phase was removed and passed through a Qiagen QiaShredder column to fragment remaining gDNA in the sample. The Qiagen AllPrep DNA/RNA 96 method was then performed including on-column Dnase 1 treatment to obtain high quality RNA with no genomic DNA contamination (Qiagen, Valencia, CA). RNA yield was determined by spectrophotometry (A260/A280) and RNA quality was determined using an Agilent 2100 Bioanalyzer (Agilent Technologies, Santa Clara, CA). The average RNA integrity number (RIN) for all 18 RNAs (3 animals times 6 tissues) was 6.4. An RNA aliquot from each organ of each animal was pooled and a total of 170 µg of RNA was prepared for sequencing.

### Library construction and 454 sequencing

The eighteen total RNA samples (6 tissues times 3 animals) were pooled equally into one pool. The total RNA pool underwent additional cleaning using the mirVana isoltation kit following manufacturer's recommendations (Ambion). Poly A RNA cDNA was synthesized according to a standard protocol using an oligo(dT)-linker primer for first strand synthesis. The N0 cDNA was PCR amplified during 18 cycles using a high fidelity DNA polymerase. Normalization was carried out by one cycle of denaturation and renaturation of the cDNA, resulting in N1-cDNA. Reassociated ds-cDNA was separated from the remaining ss-can (normalized cDNA) by passing the mixture over a hydroxylapatite column. After hydroxylapatite chromatography, the ss-cDNA was amplified with 15 PCR cycles. For 454 sequencing, cDNA in the size range of 500–700 bps was eluted from a preparative agarose gel. An aliquot of the size fractionated cDNA was analyzed on a 1.5% agarose gel. 454 adaptors were ligated to the size fractionated N1 cDNA and 3′ fragment sequenced on a Roche 454 using GS FLX technology with Titanium series chemistry following manufacturer's recommendations.

GS FLX sequencing generated 1,283,840 reads with an average length of 344 bases. Raw reads were trimmed for quality and reads shorter than 40 bases were discarded. The sequencing resulted in 1,212,395 reads of a total length 426,683,712 bases.

### Library assembly

The trimmed and filtered reads was assembled using MIRA [36] (version 3) with the following parameters: mira –job  =  denovo,est,accurate,454 454_SETTINGS -CL:qc  =  no:cpat  =  yes -AL:mo  = 40:mrs  = 90. MIRA assembly produced 62,567 contigs and 125,228 singletons. There were 85 contigs and 13,432 singletons discarded due to poor quality (repetitive or poly-T sequence) or short read length (<50 bases), resulting in 62,482 contigs and 111,796 singletons for a total of 174,278 Syrian hamster transcriptome sequences totaling 60,117,204 bases.

### Transcriptome and genome references

The transcriptome references used in this study were retrieved from the Ensembl Database [30] via the Biomart interface. Transcriptome references used in this study were obtained from the release 71 of the Ensembl database. The draft version of the CHO genome and transcriptome were retrieved from the Pre Ensembl website.

### Alignments

Syrian hamster transcriptome sequences were aligned to transcriptome references using BLAST [37]. An Expect value cutoff parameter of 10 was used and alignments results were filtered in order to only keep sequences aligned at least at 80%.

### Similarities of the assembled library with the transcriptome references

The similarities to the mouse, rat and other transcriptome references were calculated based on BLAST results. For all Syrian hamster transcriptome sequences that aligned to the transcriptomes, we calculated the ratio between the total number of correct nucleotide matches and the total combined length of our Syrian hamster transcriptome, which is 60,117,204 bases.

### Identification of over-represented canonical pathways and biological functions

Functional enrichment of canonical pathways and biological functions was performed using Ingenuity Pathways Analysis (Ingenuity Systems, Inc.). Canonical pathways refer to pathways curated by Ingenuity as part of its knowledgebase, based on extensive characterization in the peer-reviewed literature published using human, mouse, and rat experimental models. These typically represent common properties of a particular signaling module, mechanism, or pathway. IPA examines differentially expressed transcripts in the context of known biological functions, mapping each gene identifier to its corresponding molecule in the Ingenuity Pathways Knowledge Base (IPKB). For all analyses, the p-values – representing the statistical over-representation significance – were generated using the right-tailed Fisher's Exact Test [38] and were adjusted using the Benjamini-Hochberg Multiple Testing correction [39].

### Distrogram construction

The distogram represented in figure 4A was constructed using the “squash” package [40] of the R suite [41]. This representation is a color-coded, rotated triangular matrix indicating the distance between every pair of species in term of number of aligned sequences shared.

### Phylogenetic tree construction

To construct the phylogenetic tree, we used the 611 transcriptomic sequences that have been significantly aligned on the transcriptome reference of the 13 species having the largest numbers of mapped sequences and the largest degrees of shared sequences. Sequences and matched transcripts were aligned using the Needleman-Wunsch multiple alignment algorithm [42], using the multialign function in MATLAB (open and extend gap penalties have been taken into consideration). Genomic divergences between sequences were calculated using the Jukes-Cantor method [43], based on the ‘NUC44’ scoring matrix. Indel mismatches have not been taken into consideration for the computation of genomic divergences. The phylogenetic tree was constructed by using the neighbor-joining method (NJ) [44].

## Supporting Information

Figure S1 **Legend for the IPA canonical pathways representations.** Figure showing the annotations of the different node and edge shapes in the representations of the canonical pathways obtained from Ingenuity Pathway Analysis (IPA)
(PDF)

Table S1 **List of highly-covered transcripts by the Syrian hamster transcriptome.** Table showing the 214 highly-covered transcripts (transcripts mapped at least at 90% by the contigs and singletons) based on the mouse annotations. For each highly-covered transcript, the Ensembl Mouse gene identified, the associated gene name as well as the gene description is indicated.
(TXT)

Table S2 **Alignment positions of the Syrian hamster transcriptome over the Chinese Hamster Ovary cell genome.** Table showing the alignment positions of the Syrian hamster transcriptome sequences to the draft of the Chinese hamster genome. We found that 85,652 contigs and singletons of our library have been aligned on the Chinese Hamster Ovary cell genome draft. For each aligned Syrian hamster transcriptome sequence, the CHO genome segment, the start alignment position, the end alignment position, and the strand are indicated.
(TXT)

Table S3 **List of sequences used to infer the phylogenic tree.** Table providing the list of contigs and singletons used to construct the phylogenic tree shown in figure 4. All the 611 contigs and singletons in this table have been significantly aligned on the transcriptomes of species having the highest number of commonly aligned sequences.
(TXT)
