# Correction: Sequencing, Annotation and Analysis of the Syrian Hamster (*Mesocricetus auratus*) Transcriptome

**DOI:** 10.1371/journal.pone.0117958

**Published:** 2015-02-09

**Authors:** The PLOS ONE Staff

Fig. 4 is incorrect. The authors have provided a corrected version here.

**Fig 4:**
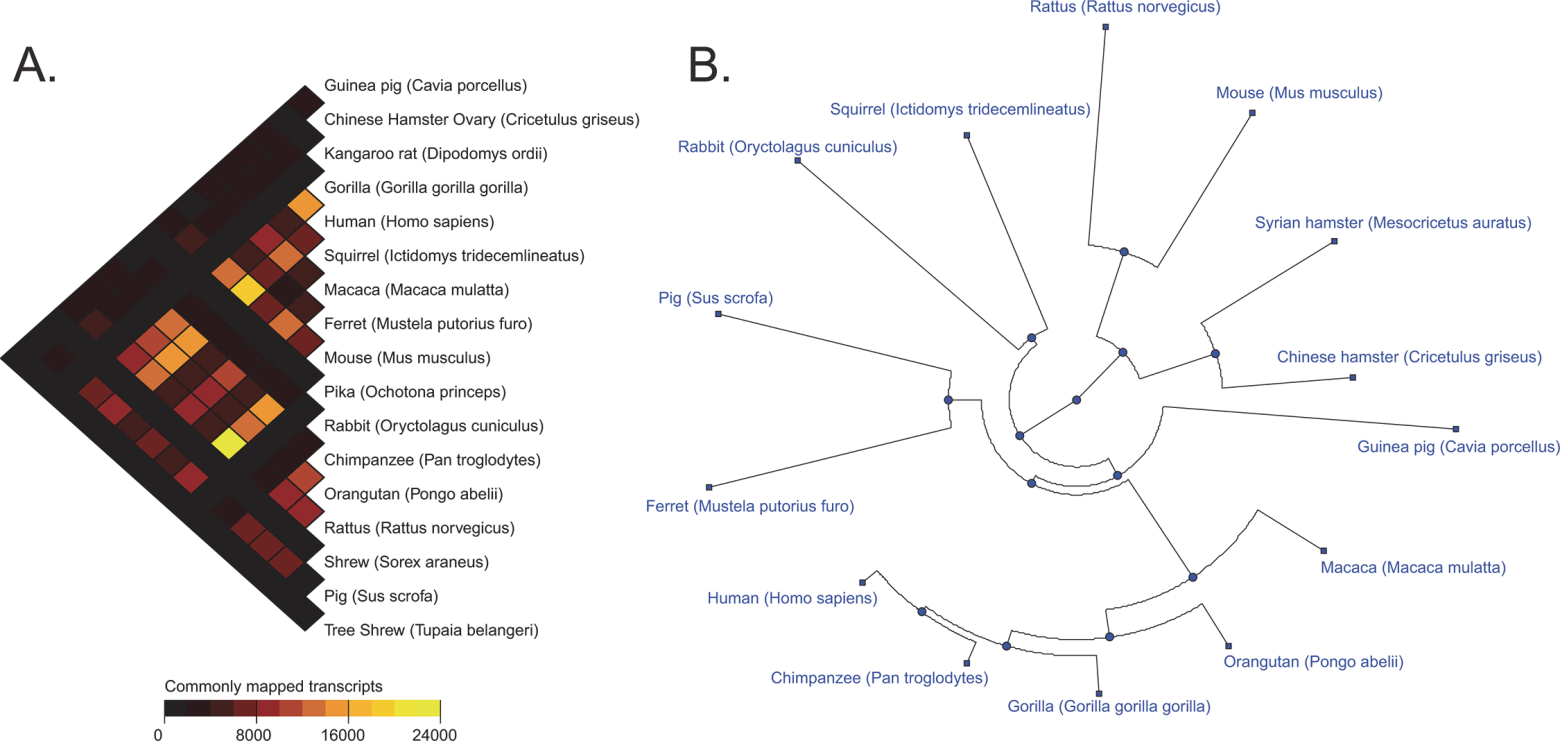
Distogram showing the commonly mapped transcripts and phylogenetic tree showing the divergences amongst the different species. (A) Distogram showing the number of transcripts commonly mapped by the Syrian hamster transcriptome between the different species used in this study. Each cell of the distogram represents the number of transcripts commonly mapped by two different species using a gradient color. (B) Phylogenetic tree showing the genomic divergence between a subset of the different species used in this study. Each leaf of the tree represents a different species and the distances of the edges are proportional to the genomic distances between the species. Genomic distances have been calculated based on the list of 611 Syrian hamster contigs and singletons that have been commonly aligned on the transcriptome references of the 13 species having the highest number of commonly aligned sequences. doi:10.1371/journal.pone.0112617.g004
