# Supplementary figures and images for: Sequencing, Annotation and Analysis of the Syrian Hamster (Mesocricetus auratus) Transcriptome
Source: PLoS One. 2014 Nov 14;9(11):e112617. doi: 10.1371/journal.pone.0112617 (PMC4232415; doi:10.1371/journal.pone.0112617)

# SUPPLEMENTARY FIGURE 1

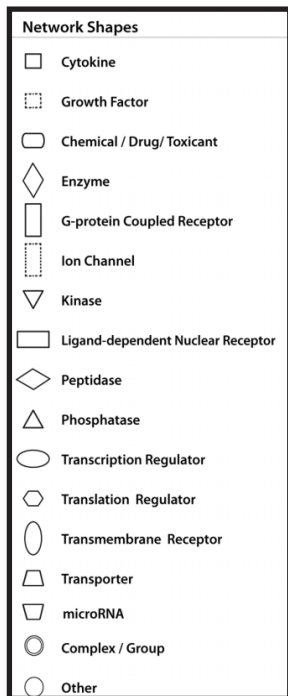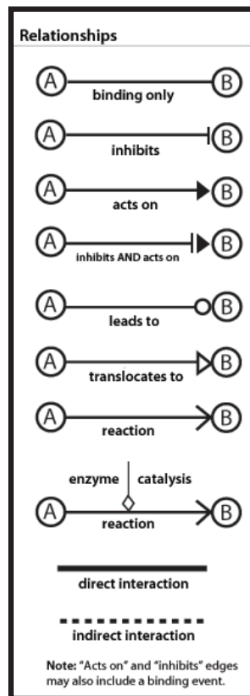

Supplement: Figure S1 — Legend for the IPA canonical pathways representations. Figure showing the annotations of the different node and edge shapes in the representations of the canonical pathways obtained from Ingenuity Pathway Analysis (IPA) (PDF) [file pone.0112617.s001.pdf]
